# Navigating diagnostic uncertainty in children's chronic lower limb pain: A qualitative study of management strategies using vignette‐based focus groups

**DOI:** 10.1002/jfa2.70032

**Published:** 2025-03-05

**Authors:** Jessica Coventry, James J. Welch, Verity Pacey, Binh Ta, Elizabeth Sturgiss, Mitchell Smith, Cylie M. Williams

**Affiliations:** ^1^ School of Primary and Allied Health Faculty of Medicine, Nursing and Health Sciences Monash University Frankston Victoria Australia; ^2^ Ablefeet Ltd. Surrey UK; ^3^ Department of Health Sciences Faculty of Medicine, Health and Human Sciences Macquarie University Macquarie Park New South Wales Australia

**Keywords:** children, chronic pain, diagnostic uncertainty, focus groups, podiatry

## Abstract

**Background:**

Chronic lower limb pain is common in children and adolescents and is frequently managed by podiatrists. Due to the complexities of understanding the cause of chronic pain, clinicians may experience uncertainty around the diagnosis, which in turn may impact their communication and management approaches. Limited research explores how podiatrists manage chronic lower limb pain in children, especially in the presence of diagnostic uncertainty. This study aimed to explore the management strategies including language that podiatrists report using to address the pain experience of children with chronic lower limb pain and to investigate if and how the reported management strategies used by podiatrists to address the pain experience of children with chronic lower limb pain vary based upon the level of diagnostic uncertainty.

**Methods:**

Eight focus groups were conducted with a total of 48 podiatrists. Participants were presented with three vignettes, each describing a child with chronic lower limb pain. They were then asked to discuss their certainty in the child's diagnosis presented and their approaches to explain and manage the child's pain. Audio data were recorded, transcribed and analysed using thematic analysis. Three key themes were generated: Language strategies, non‐verbal communication strategies and treatment strategies.

**Results:**

Podiatrists were overall certain in the diagnosis presented in vignettes 1 (calcaneal apophysitis) and 2 (juvenile idiopathic arthritis); however, they expressed significant uncertainty in vignette 3, which was written to elicit uncertainty presenting a case with generalised lower limb pain. Many groups fixated on the Beighton score of 5/9 and interpreted this to mean hypermobility, which is inconsistent with the current clinical guidance. Podiatrists used similar language strategies across all 3 vignettes and supported their language strategies with non‐verbal communication strategies. Podiatrists also discussed activity modification, passive and self‐care strategies and building a team as the treatment strategies they would use.

**Conclusions:**

This study highlights the variety of clinical management strategies used by approaches and highlights how their approach may change depending on their certainty in the diagnosis.

## 1 INTRODUCTION

Children's chronic pain presents many challenges in its diagnosis, assessment and management. Chronic pain is defined as pain that persists or recurs for more than three months [1]. It is estimated that, globally, 1 in 5 children and adolescents experience chronic pain [2]. For children, chronic pain, especially in the lower limb, has significant impacts including decreased school attendance, social participation and physical function [3, 4, 5, 6, 7]. This often leads to decreased physical activity and other health issues, as well as overall higher levels of anxiety, depression and decreased quality of life [3, 4, 5, 6, 7]. In addition to the impacts on children's own lives, chronic pain has significant negative consequences for their families. Families of children with chronic pain report increased anxiety, parenting stress, financial burden and job instability due to care responsibilities impacting their work [8, 9, 10]. For both children and their families, these consequences are increased when pain is poorly controlled or when the cause of the pain is unknown [11, 12].

Chronic pain is commonly a feature of medical conditions with greater disability impact, such as pain associated with cerebral palsy or juvenile idiopathic arthritis (JIA). However chronic pain may also occur without another explanatory chronic condition or diagnosis [1], defined as chronic primary pain in the ICD‐11, which includes conditions such as generalised musculoskeletal pain [13].

These variations in the causes of chronic pain, in addition to the many biopsychosocial factors that contribute to the pain experience, result in significant complexity for health professionals and families. Chronic pain with diagnostic uncertainty often leads to delays in both referral and diagnosis [14] and results in distress for families [15]. Even after a working diagnosis and management plan, families may continue searching for alternative explanations for the persisting pain [15].

Diagnostic uncertainty also has a significant impact on health professionals. Health professionals such as paediatricians and physiotherapists describe feelings of doubt and anxiety while information gathering, problem‐solving and communicating in the presence of diagnostic uncertainty [16, 17]. This then impacts the development of management plans. This is especially common in health professionals who treat children with chronic pain [17, 18].

Pain education is a vital part of the management of chronic pain. However diagnostic uncertainty may inhibit effective pain education without the knowledge of a definitive mechanism for the pain [19]. Ineffective pain education may further uncertainty and mistrust of health professionals, which may have a cyclic effect of seeking health services, and receiving inconsistent information about their pain, worsening the outcomes [15].

The impact of diagnostic uncertainty has been explored among children with chronic pain, their families and clinicians [15, 18]. However, given the prevalence of diagnostic uncertainty in children's chronic pain, it is important to also understand how diagnostic uncertainty impacts clinical management where chronic pain is a feature of the presentation. Given their foot and lower limb expertise, podiatrists are commonly involved in the diagnosis and management of chronic lower limb pain. However, we were unable to find studies specific to podiatrists and diagnostic uncertainty in paediatrics.

Therefore, the primary aim of our study was to explore the management strategies including language that podiatrists report using to address the pain experience of children with chronic lower limb pain. Furthermore, to investigate if and how the reported management strategies used by podiatrists to address the pain experience of children with chronic lower limb pain vary based upon the level of diagnostic uncertainty.

## 2 METHOD

### 2.1 Study design

This was a qualitative research using vignette‐based focus group discussions. This design was selected to facilitate discussion and knowledge sharing as a social interaction among diverse participant perspectives [20, 21].

A social constructivism theoretical orientation was used throughout this study. This was underpinned by the idea that knowledge develops through interactions that individuals have with one another. Social constructivism is both an ontology and epistemological positionality, where multiple realities exist and are constructed through lived experiences and interactions with others [22].

We used the criteria of the Consolidated Criteria for Reporting Qualitative Research (COREQ) guidelines [23] to guide the conduct and reporting of our findings.

### 2.2 Participants and recruitment

Participants were recruited during the 2023 Australian Podiatry Association conference in Brisbane, Australia. This bi‐annual conference is run over three days, with an estimated 1000 podiatrist delegates. Recruitment to attend an education and research focus group forum was carried out through the conference organisers on behalf of the research team using the workshop abstract. This research and the opportunity to pre‐read the explanatory research statement were advertised within the conference programme app, and during plenary speaking sessions through a QR code placed on the screen for conference delegates. All podiatrists attending were eligible to participate using a convenience sampling method to recruit.

### 2.3 Data collection

Potential participants attended a 90‐min workshop on the final day of the conference. Three authors co‐facilitated the workshop (JC, MS and CMW). JC (physiotherapist) and MS (podiatrist) are both PhD candidates, and CMW (podiatrist) is an experienced researcher with paediatric education and research experience.

At the commencement of the workshop, the team explained the education structure and research component, and participants were given time to review the explanatory statement, ask questions of the team and provide written informed consent. The workshop room was set up with both recording and non‐recording tables, therefore, if workshop participants decided not to participate in the research, they were still able to fully engage in the workshop. Tables were set up to allow up to 10 participants per table.

The workshop commenced with a 10‐min introduction to the concept and impact of children's chronic lower limb pain and the aims of this research. As part of consent, participants also responded to questions about age, gender identity and qualification. Data were collected through a semi‐structured focus group format with three specific questions aligned to three vignettes.

### 2.4 Vignettes

The vignettes used in this study were developed by the research team from teaching resources available to the podiatric medicine programme at Monash University. All three vignettes were first‐person narratives of children or their parents describing a child with chronic lower limb pain with varied diagnostic uncertainty. In vignette 1 enough information was provided for podiatrists to be sure of their diagnosis, in vignette 2 podiatrists were given an explicit diagnosis and in vignette 3 no explicit diagnosis was given. All the children in the vignettes were between 11 and 12 years of age (Table 1). In vignette 1, calcaneal apophysitis was used as a common condition we considered podiatrists likely to be familiar with in terms of both diagnosis and management. In vignette 2, juvenile idiopathic arthritis (JIA) was chosen as a condition and we also considered that podiatrists would be familiar with diagnosis and management strategies; however, the pain profile was more complex, and podiatrists may be less confident in its management. For both vignettes 1 and 2, we anticipated that podiatrists would have an understanding of common podiatric conditions of the lower limb from their training regardless of their current clinical practice [24].

**TABLE 1:** Vignette summaries.

Vignette	Age	Condition	Summary
1: Archer	11	Calcaneal apophysitis	● Loves soccer, previously played on a team ● Recently tried to play again but it was too sore ● Heel pain since last year ● Mum thinks it could be “Severs disease” ● Worried this ‘disease’ might be contagious ● When he runs it gets more painful and it makes him walk funny
2: Tamar	11	JIA	● Passionate Australian Football (AFL) player ● Is unable to play in his usual position due to too much jumping and some weeks he is too sore to play ● Walked funny in the mornings and felt stiff in his ankles and knees ● Had blood tests and needles ● Doctor said he has JIA, an arthritis that kids get ● He limps less now from the tablets and needles but is still in pain ● Does not want to tell his mum about his pain because she might stop him from playing
3: Ellie	12	Generalised non‐specific lower limb chronic pain	● Has had pain since she was 6 ● Quiet and would prefer to stay inside during lunch time ● Extremely tired after school ● Wakes up at night wanting her legs rubbed, sometimes she wakes up screaming in pain. ● X‐rays and blood tests say there is nothing wrong ● Rheumatologist found Beighton score of 5/9 ● Mum cannot remember the last time Ellie or the family had a full night sleep ● Ellie is about to start high school

Finally, vignette 3 was designed to portray a ‘growing pains’ or primary musculoskeletal pain case where testing was completed to rule out sinister conditions; however, non‐specific chronic pain remained. This type of pain can prove challenging for clinicians as there is no consistent aetiology that results in growing pain [25]. Table 1 provides the vignettes provided at each stage of the focus group. Detailed vignettes that were provided to participants are in Appendix 1.

The vignettes were presented in the order outlined in Table 1. At each stage, the vignette was provided on the screen and paper to participants, and participants were asked to describe their certainty in the diagnosis, as well as the clinical management strategies they would use for the child's pain. Participants had between 10 and 25 min to discuss each vignette.

The facilitating researchers (CMW, JC and MS) drifted around the room and recorded key phrases and words from tables which were transcribed by a graphical recorder to create an initial concept map providing continual feedback to the participants as the workshop progressed. The initial graphical recorder sketches are presented in Appendix 2.

### 2.5 Data analysis

Following the workshop, the concept map was sent out as a resource to all workshop attendees and reviewed by the research team before commencing coding of the focus group transcripts. Group audio recordings were uploaded into auto‐transcribing software Otter.ai (AlSense 2016). Transcriptions were manually checked and the identifying information of participants was removed by the first author (JC). Due to the nature of the recording of multiple voices without identifiers, demographic data were not linked to responses.

Thematic analysis was used to analyse the data as outlined by Braun and Clarke [26]. The process of transcription correction allowed the first author (JC) to become familiar with the data. Corrected transcripts were read and re‐read by the first author. Two authors independently coded all transcripts (JC and JJW). These codes were tentatively clustered into potential themes by JC, before being reviewed by the full authorship team. In an iterative discussion with the full team, the themes and subthemes were revised and further defined into the final themes.

One author (JC) then used descriptive analysis to map the level of diagnostic uncertainty across the vignettes by analysing the transcripts. This guided the interpretation of the strategies which were used in differing levels of diagnostic uncertainty.

## 3 RESULTS

### 3.1 Demographics

A total of 54 podiatrists attended the workshop, of these, 48 podiatrists consented to participate at the recorded focus group tables. Podiatrists who chose not to participate in the study were not asked the reason for their decision not to participate. Eight focus group tables were recorded, and each group had 3–9 podiatrists and participant demographic characteristics are displayed in Table 2.

**TABLE 2:** Participant characteristics.

Demographic	Category	Participants
		*n* (%) or mean (SD), range
Gender	Woman	39 (81%)
	Man	9 (19%)
Age (years)		38 (11), 23–61
Highest qualification	Bachelor	36 (75%)
	Masters	6 (13%)
	PhD	4 (8%)
	Other –(associate Diploma)	2 (4%)
Number of years of registered		11 (8), 0.5–34
Average % of caseload pediatrics	0%–20%	25 (52%)
	21%–40%	15 (31%)
	41%–60%	3 (6%)
	61%–80%	0
	80%–100%	3 (6%)
	Not completed	2 (4%)

### 3.2 Management strategies for children's chronic pain

**FIGURE 1:**
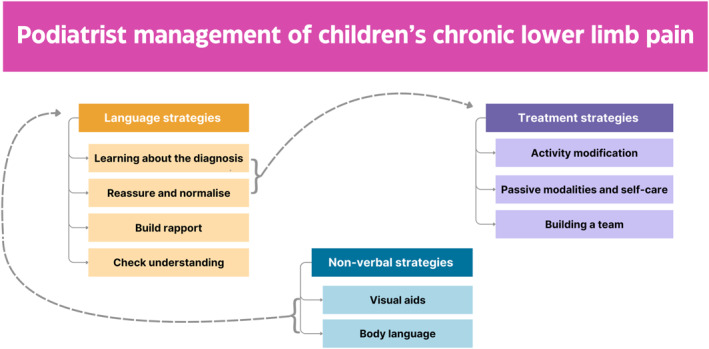
Podiatrist management of children's chronic lower limb pain.

Podiatrists drew on a variety of *language‐based and non‐verbal communication strategies* and various *treatment strategies* when discussing the clinical management of the children presented in the vignettes (Figure 1). Illustrative data for these strategies are presented in Table 3. Podiatrists underscored the importance of good communication as a management tool for chronic pain. They discussed strategies such as *teaching the child and their family about their diagnosis*, *normalising and reassuring*, *checking the child's understanding of their pain or condition* and *building trust and rapport*. Podiatrists also discussed how they might support these language‐based strategies with non‐verbal strategies by using *visual aids* or adjusting their *body language* to be more open and to allow them to speak to the child at their level. Additionally, this analysis identified three key treatment strategies that podiatrists drew on. *Activity modification, passive modalities and self‐care* and *building a team.*

**TABLE 3:** Illustrative data clinical management strategies.

Theme	Subtheme	Vignette	Supporting quotes
Language strategies	Learning about the diagnosis	Vignette 1	*“I say straight away it's not a disease”—Group 4*
		Vignette 2	*“If they go, what is JIA? And you go well its juvenile idiopathic arthritis, and they go well what is idiopathic?” —Group 8*
		Vignette 3	*“I talk about pulling a basket out of a well. So if you've got a rigid rope, which is like your normal muscle, you can pull the bucket out of the well. But if you've got a really elastic rope, you actually have more tension on it, so you have to work so much harder, just to pull the same bucket out of the well.”—Group 2*
	Normalise and reassure	Vignette 1	*“I also like to reassure them that this is a common thing”—Group 3*
			*“I usually say it's related to their growth or something like that…this is something that they'll grow out of as well. It's not forever”—Group 3*
		Vignette 2	*“Explain that they went through some rehab and they've stopped playing for a little bit and got stronger and look at them, they're back playing”—Group 4*
		Vignette 3	*“you've obviously seen a number of people at this point that also aren't quite sure what's going on it doesn't know when that your symptoms, or that you know, what you're experiencing isn't valid or real, it just means that we don't quite know and sometimes we don't always have the answers”—Group 6*
			*“I like to reassure as well in terms of, you know, sometimes being told by [a] rheumatologist that they had a score but not explaining what that is…So you can sort of say, hey, you know what, there's actually a lot of the people in the population that are hypermobile”—Group 1*
	Building rapport	Vignette 1	*“Share a little bit of a story about yourself but I had this when I was your age, I was playing footy.”—Group 7*
		Vignette 2	*“Tell me five things you love. You've got Minecraft and soccer, what else?”—Group 8*
		Vignette 3	*“Being completely transparent when you are not sure… I think that's also so important for building rapport with parents, kids, patients in general.”—Group 6*
			*“We really want to know [about you] and we really want to help you minimize that pain.—Group 1*
	Check understanding	Vignette 1	“*Ask them their understanding of pain.”—Group 4*
		Vignette 2	*“we'll see whether the patient or their mum actually understands what that means and how it affects them.”—Group 3*
		Vignette 3	*“I want her to explain it to us first because we don't know what it is”—Group 7*
Non‐verbal strategies	Visual aids	Vignette 1	*“I normally will bring up an X‐ray or some google images”—Group 6*
			*“I actually bring up a paediatric X‐ray and I go, this is a calcaneal bone. That's where this part of the word comes from. And this is the little bit this is the apophysis that we're looking at. Yeah. And this is what it looks like on you. And then I bring up however old mum is and I said, this looks like on mum it looks different. And that can they can kind of then rationalize, this is what my body can look like”—Group 2*
		Vignette 2	*“x‐rays and what they look like or showing pictures [or] kids drawing pictures of their pain and what it looks like to them”—Group 4*
	Body language	Vignette 3	*“Being mindful of your body language… making sure you're at their level, like not standing up when they're sitting down and trying to maintain eye contact, being really close, I think, yeah, making them feel sort of relaxed and empowered in that situation”—Group 1*
Treatment strategies	Activity modification	Vignette 1	*“You don't need to stop but we do need to just dial that down a bit.”—Group 4*
			*“slow your activity down or stop your activity for a short period of time, when we treat it successfully, it will get better. It is self‐resolving.”—Group 7*
		Vignette 2	*“Listen to your body, they're the sort of words that I would use and say listen to your body. And it's absolutely okay to keep playing, you know, this condition shouldn't hold you back. But if you do have a day, when you're having a worse day than another, it's okay to take a day off and you can come back stronger the next day”—Group 1*
			*“They might have other things that they really enjoy that we can look into or that we can modify”—Group 3*
			*“The aim is not to stop [you] moving. If anything, it's to keep you moving in comfort.”—Group 1*
			*“Motion is lotion”—Group 8*
		Vignette 3	*“ Pacing and so understanding and respecting that her body might, we might need to then understand that there might be periods of rest and that's okay and there's a respect of the body. But over time, if you can then compound capacity, you'll be able to do more for longer.”—Group 4*
			*“Activity she can do where she doesn't realise, she's doing activity like it might be gardening… or cooking”—Group 3*
	Passive modalities and self‐care	Vignette 3	*“She needs some empowering self‐management strategies that she can employ in the middle of the night without waking up at parents”—Group 7*
			*“I use a toolbox method. So I say, we're going to put some of these ideas into a toolbox. And you can take things…out of your toolbox in any time.”—Group 2*
	Building a team	Vignette 2	*“I would also like to involve the school in this pain as well”—Group 2*
			*“I think Mum needs lots of support. And it's okay to ask for help. Because it's a really tough thing that the whole family's going through.”—Group 5*
		Vignette 3	*“OT and an exercise [physiologist] for the low tone stuff like that”—Group 5*
			*“I find that I will draw like a triangle… at the top of the triangle it's you, you're in charge. The podiatrist's role is sometimes when your joints get sore our job is trying to protect your joints if we can by our means. And then the doctors role or rheum [sic] role is to make sure that everything is optimised and moving forward and if were all doing everything we can… not technically to the child but maybe to the parents”*

These language‐based, non‐verbal treatment strategies were not used in isolation, instead, podiatrists drew on multiple strategies and used them to support one another. Figure 1 maps how these strategies were used in combination with one another. For example, for vignette 1, some groups discussed how they would reassure and normalise Archer about his calcaneal apophysitis while discussing how his activities could be modified day to day.

### 3.3 Diagnostic uncertainty across the vignettes

Podiatrists described different levels of uncertainty across the three vignettes with examples of comments provided in Table 4.

**TABLE 4:** Diagnostic certainty illustrative data.

Vignette	Interpretation	Supporting quotes
Vignette 1 (Archer)	Podiatrists were certain of the diagnosis of calcaneal apophysitis	*“I think quite certain in the diagnosis but still want to do an assessment, it's not very descriptive at all.” (Group 1)*
		*“Yeah so its right age, right activity. Flares up with rapid movements. So yeah I'd be pretty confident in the diagnosis there.” (Group 3)*
		*“One hundred percent” (Group 3)*
		*“I was gonna say, it sounds pretty stock standard” (Group 5)*
		*“I think the diagnosis fits the age group.” (Group 6)*
Vignette 2 (Tamar)	Podiatrists were certain of the diagnosis of JIA	*“Ok for the diagnoses considering we've had support from a medical team as well, we've had blood tests and things like that, and we're in good communication with them, I think we can be relatively certain of it yeah.” (Group 1)*
		*“You'd have to assume that the blood tests are right.” (Group 5)*
		*“it's very likely a rheumatologist by the sounds of that with all the tablets and needles. So I would be pretty confident that diagnosis is correct.” (Group 7)*
Vignette 3 (Ellie)	Podiatrists were uncertain about Ellie's diagnosis in vignette 3	*“Zero certainty in the diagnosis” (Group 6)*
		*“Definitely not confident in the diagnosis because there isn't a diagnosis.” (Group 2)_*
	Podiatrists considered how much other factors such as hypermobility may contribute to Ellie's pain	*“I'm not certain in this diagnosis at all. I'm not certain in this diagnosis. I know they said they've done all the tests. So I assume they don't have low tone but generally when we get these kids that are hypermobile, they might have some degree of low tone as well, which means that they get tired more easily.” (Group 3)*
		*“I mean, the [Beighton score] is five out of nine might infer that she's her muscles are working very hard to stabilize the joints which is fatiguing her.” (Group 1)*
		*“You'd have to be worried you've got, you know, they're stretching… well they're hyper mobile. Potentially, there's some sort of joint condition going on. Bit of muscle stuff. No sleep.” (Group 5)*
		*“Because it might actually be, like some of this, some of this could also be maybe a little bit of neurodivergency.” (Group 2)*
		*“If she never liked sport, does she have an underlying like DCD condition as well that we haven't explored? She's never seen physio she's only seen is it GP? So that could be definitely worried about genetics, Developmental Coordination Disorder” (Group 3)*
		*“And you could probably assume there's probably some headaches in there. With those kids as well lots of headaches.” (Group 5)*
		*“I wonder if she's a fussy eater” (Group 5)*
	Some groups redirected their groups to focus on managing Ellie's pain rather than seeking a cause	*“a diagnosis isn't going to change the pain.” (Group 5)*
		*“We're not searching for a diagnosis, we're trying to help Ellie get to where she wants to be” (Group 1)*

Overall, podiatrists expressed certainty in the diagnoses in vignettes 1 and 2. In vignette 1 podiatrists discussed familiarity with the presentation, pathophysiology and management of calcaneal apophysitis. In vignette 2, podiatrists' certainty in the diagnosis of JIA was supported by the investigations completed by the medical team, particularly blood work. In vignettes 1 and 2 all eight groups reported elements of certainty in the diagnosis. Conversely, in vignette 3, many podiatrists expressed uncertainty in the cause and therefore diagnosis relating to Ellie's pain. Podiatrists often started their vignette discussion with *“very uncertain”* (group 7) or *“I'm not certain in this diagnosis at all.”* (group 3). Although some remained uncertain, some groups began to discuss contributing factors and possible causes of Ellie's pain. These discussions appeared to increase their confidence in their diagnosis: *“definitely hypermobility”* (group 4); however, the potential causes they pursued were not necessarily indicated within the vignettes provided (Table 4).

Podiatrists who indicated diagnostic uncertainty discussed other possible causes of pain that were outside of the information included in the vignette. Some podiatrists were able to redirect their group away from pursuing alternative explanations of a diagnosis by highlighting that a diagnosis would not change the way they needed to manage Ellie's pain.

Podiatrists used similar language strategies across all 3 vignettes and supported their communication with non‐verbal strategies. However, the way they used them differed slightly across vignettes 1 and 2 when compared to vignette 3. For example, when they were certain of the diagnosis in vignettes 1 and 2, podiatrists discussed using visual aids such as foot models, drawings and X‐rays to support their language strategies when providing pain and condition‐specific education to children and their families. Conversely, in vignette 3, there was no clear diagnosis and therefore no clear pathophysiology that they could use their visual aids to explain. Instead, podiatrists discussed how they could use their body language to establish rapport with and reassure the child.

Podiatrists talked about activity modification for all 3 vignettes. However, this was not the case for the other management strategies: ‘Passive modalities and self‐care’ and ‘Building a team’. Instead podiatrists primarily focused on building a team for vignettes 2 and 3. Perhaps due to the ongoing nature of the pain in the presentations in these vignettes in comparison to Archer's calcaneal apophysitis in vignette 1. Passive modalities and self‐care were almost exclusively discussed during vignette 3, where podiatrists discussed strategies, they could provide to Ellie to assist her with building independence in the management of her pain.

## 4 DISCUSSION

To our knowledge, this is the first study known to explore podiatrists' diagnostic uncertainty and chronic pain management in children's chronic pain. The findings demonstrate that podiatrists use a variety of strategies including language‐based and non‐verbal communication strategies and various treatment strategies.

Podiatrists used similar language strategies irrespective of the level of diagnostic certainty in the vignettes; however, they did discuss differences in their use of visual aids. In vignettes 1 and 2 where podiatrists were certain of the diagnosis, they used visual aids such as x‐rays and drawings to explain the child's pain and orientate them to relevant anatomy. However, in vignette 3 podiatrists instead discussed how they would adjust their body language by getting down on the patient's level in combination with their language strategies to build rapport and reassure the child. Our findings align with recommendations for child‐centred communication during consultations for chronic health conditions [27]. However, understanding a child's concept of pain may also assist the clinician with tailoring their pain education [28]. Additionally, using visual aids may help increase engagement as well as understanding, particularly in younger children [27].

The uncertain diagnosis in vignette 3 provided a challenge to design a management programme. At times, podiatrists extrapolated from small pieces of information within the vignette leading to conclusions that were not necessarily indicated. Hypermobility was frequently discussed and focused on during management discussions. Most groups interpreted Ellie's Beighton score of 5 out of 9 to indicate that she had joint hypermobility. This is despite clinical guidance that a clinical diagnosis of generalised joint hypermobility in children requires a Beighton score of ≥6 [29]. Although the focus group methodology opens itself up to biases created by ‘group think’, this does highlight that podiatrists may not be up to date on contemporary evidence‐based recommendations for identification of generalised joint hypermobility. Interestingly several podiatrists were able to redirect their group from pursuing a diagnosis as they felt it would not change the management of the child's pain. Podiatrists spoke about being comfortable with not knowing and being honest with the child and their family. A 2023 study of paediatricians treating children's chronic pain similarly found that paediatricians felt that being honest and open about their uncertainty was helpful and appropriate [17]. However, children and their families may find this unhelpful, and clinician uncertainty can instead increase mistrust and fuel their anxieties that something is being missed [15, 30].

Across all three cases, it was clear that podiatrists were focused on activity modification that allowed for as much physical activity as tolerated within pain limits. Movement is a key component of pain management [31, 32, 33]. For children, sports and activities are often the way that they socialise and limiting participation in activities may also impact their ability to maintain friendships and can lead to isolation and loneliness [34, 35]. In vignettes 1 and 2, activity modification was a key part of podiatry management. In vignette 3, where there was diagnostic uncertainty, in addition to increasing Ellie's physical activity levels, podiatrists focused on brainstorming strategies and tools that could be added to Ellie's ‘toolbox’. The pain toolkit method was conceptualised and popularised by Pete Moore, originally for people living with fibromyalgia [36]. This method focuses on giving individuals strategies and education that allow them to self‐manage their pain [36].

Building a multi‐disciplinary team and a supportive community was another major focus of discussion for vignettes 2 and 3. The diagnosis of JIA in vignette 2 and lack of diagnosis but generalised musculoskeletal pain in vignette 3 likely indicated the need for ongoing support. Podiatrists discussed needing to build a team of healthcare professionals around the child. This team included physiotherapy, occupational therapy, exercise physiology, rheumatology, dietetics and psychology. Existing literature emphasises the benefits of a multi‐disciplinary approach to chronic pain management in children [30, 37]. In addition to building this team around the child, podiatrists also considered psychological support and referrals for the child's parent. Podiatrists identified that in both vignettes there was an element of fear and anxiety reported by the child's parent. Parents of children with chronic pain report problems with coping and feeling restricted in their own social lives [8, 38]. Studies suggest that parent anxiety can lead to increased pain catastrophising and protective behaviours which can reduce children's function [39]. Additionally, parents play a key role in helping children navigate their chronic pain journey and encouraging good pain management behaviours [40, 41].

A key strength of this study is its design, a qualitative focus group methodology allowed for the sharing and evaluation of strategies by podiatrists. Additionally, the vignette‐based approach allowed for discussion to be structured around real‐life contexts and encouraged podiatrists to reflect on their own practices to understand how they might apply their knowledge. The narrow data collection context provided by the conference setting may be considered a limitation of this study. However, equally, the podiatrists involved in this study had, over the course of the last three days, been exposed to the latest evidence and practices which may have led to more informed responses. Another limitation of this study is that most podiatrists involved in the study had limited paediatric experience, which may have influenced the findings. Furthermore, podiatrists were not blinded to the fact they were participating in a study and may have selected strategies for discussion that they believed to be best practice which may not reflect their usual professional practice. Despite these limitations this study offers valuable insights into how podiatrists manage children's chronic lower limb pain, especially in the context of diagnostic uncertainty.

## 5 CONCLUSION

This study highlights management strategies used by podiatrists when managing common presentations of children's chronic lower limb pain. Additionally, it highlights how diagnostic uncertainty may impact management. In familiar conditions, podiatrists were comfortable providing clear communication and condition‐specific education; however, in the presence of diagnostic uncertainty podiatrists emphasised building rapport and children‐centred care. Podiatrists underscored the importance of activity modification and building a team to support children with ongoing chronic pain. They supplemented these with passive strategies, and self‐care options that children could use by themselves increasing their independence in managing their pain.

## AUTHOR CONTRIBUTIONS

**Jessica Coventry:** Conceptualization; methodology; investigation; formal analysis; project administration; visualisation; data curation; writing—original draft; writing—review and editing. **James J. Welch:** Formal analysis; data curation; writing—review and editing. **Verity Pacey:** Conceptualization; writing—review and editing, visualization, supervision, funding Acquisition. **Binh Ta:** Conceptualization, methodology; supervision; writing—review and editing. **Elizabeth Sturgiss**: Conceptualization; methodology; writing—review and editing, supervision, funding Acquisition. **Mitchell Smith:** Investigation, writing—review and editing. **Cylie M. Williams:** Conceptualization; methodology; investigation; resources; data curation; supervision; writing—review and editing; funding acquisition.

## CONFLICT OF INTEREST STATEMENT

CMW is an associate editor of the Journal of Foot and Ankle Research. It is journal policy that editors are removed from the peer review and editorial decision‐making process for the papers that they have co‐authored. All other authors declare that they have no competing interests.

## ETHICS STATEMENT

Approval was given by the Human Research Ethics Committee of Monash University (Approval number 37613). All participants provided consent to being part of this study.

## PERMISSION TO REPRODUCE MATERIAL FROM OTHER SOURCES

Written permission has been provided for the inclusion of artwork by Patch Creative used in Appendix 2.

## CLINICAL TRIAL REGISTRATION

N/A.

## Supporting information

Supplementary Material 1

Supplementary Material 2

## Data Availability

All summary data that supports the findings of this study are in this article and supplementary files. The individual data transcripts are not publicly available due to ethical restrictions.
