# Supplementary material for: Navigating diagnostic uncertainty in children's chronic lower limb pain: A qualitative study of management strategies using vignette‐based focus groups
Source: J Foot Ankle Res. 2025 Mar 5;18(1):e70032. doi: 10.1002/jfa2.70032 (PMC11881608; doi:10.1002/jfa2.70032)
Supplement: Supplementary file 1 — Supplementary Material 1 [file JFA2-18-e70032-s002.pdf]

Vignette 1

My name is Archer and I'm 11 years old. I'm in grade 5 at school. I like playing with my friends at lunch time and on the weekend, I used to like playing soccer with my team. I also used to practice soccer with my dad and little brother HEAPS.

At the end of last year my heels got really sore and my mum googled it and said it sounded like Sever's Disease. This made me worried I'd give it to my little brother. She took me to the doctor who told me to stop playing soccer for a while. The pain stopped pretty quickly. I played a lot of Minecraft. I wish I got out of school instead of soccer, but my heels didn't really get too sore at school

I went back to soccer after a few weeks of my heels not being sore, but they got sore again so I wanted to stop. Mum wouldn't let me so I kind of played a bit, but not like I used to. I was getting puffed really easy and the coach yelled at me to go faster. Whenever I run fast, my heels get really sore again and it makes me walk funny. My little brother doesn't seem to have seemed to caught it, so maybe it's just something wrong with me?

It's now the middle of the year. My heels have been sore since last year and mum is taking me to see a podiatrist/physiotherapist because I want to give up soccer. What's the use in going if I can't keep up with all the other kids.

<u>Questions</u>

How certain are you in the diagnosis of this condition?
How would you explain the pain to Archer and his family?

> ***(Consider What phrases, words and analogies would you usually use in this situation? Describe any non-verbal strategies would you use?*** **How would you discuss daily impact or the future?)**

What management strategies would you recommend for Archer's pain?

@Monash University, 2023

Vignette 2

My name is Tamar. I'm 11 years old and I live for AFL. My team is the Gold Coast Suns. We're not doing that well, but I go to every match I can, love kick to kick at the end of the game, and play on the weekends too. I used to play in the ruck when I was younger, but now I stick to full forward so I don't have to run or jump so much anymore. Some weeks I can't play though.

When I was 8 I had a great season. I even played reps. But I was always walking funny after a game. Then I'd be walking funny after training, and within a few months I was just walking funny all the time, but it was the worst in the mornings after I'd been sleeping. Mum said I had a limp, I said my knee and my ankle would get sore and I just felt stiff some days. Mum was too busy to take me to the doctor and just tried to get me to rest a bit, but it didn't help. Then one day she noticed that my joints were all blown up big - my tracksuit pants couldn't even go on over my knee. She took me to the doctor then and seemed really worried.

The doctor poked and moved my legs. I had to have blood tests. They really hurt. I used to hate needles, but I'm used to them now. They said I had JIA. It's arthritis that kids get. And that's when things started to change. At first I had to have a lot of tablets, and some needles in my knee and ankle, but I started walking a bit better and mum and the doctor seemed happy. I wasn't, the pain didn't change much. Then I had to have medicines in hospital via a needle. I spent a lot of time there for a while and the pain got a bit better.

Now, the medicines are just what I do. The tests are part of what I do too. We see the doctor every few months and I follow on with whatever she and mum say. I don't really limp anymore, I only sometimes feel stiff, but the pain is still there in my knee and my ankle. When we go to watch the Suns play I always do kick to kick though. Always, no matter how sore I am. I don't tell mum I'm sore - otherwise she wouldn't let me, and she'll get worried again too. I don't really tell her about the pain much because I know it makes her sad.

<u>Questions</u>

How certain are you in the diagnosis of this condition?
How would you explain the pain to Tamar and his family?
(Consider What phrases, words and analogies would you usually use in this situation? Describe any non-verbal strategies would you use? How would you discuss daily impact or the future?)
What management strategies would you recommend for Tamar's pain?

@Monash University, 2023

Vignette 3

My name is Laura, and my daughter's name is Ellie. Ellie had a hard time when she was 6, moving from kindergarten to school full time. I think a lot of her difficulties were because of leg pain. Ellie has always been pretty quiet,, not like her older sisters. She would prefer to sit down and play at lunch time and always hated sport at school. She made a few friends when she was younger but always said they would run off at school and play but she couldn't keep up. I always made her do swimming on the weekend, but she never did sport after school because she is just so tired after school.

Ellie has woken up at night wanting her legs rubbed since she was 5. Sometimes she screams so loud it wakes the house up so we have to get to her quickly. We have taken her to the GP and a Rheumatologist. Both tell us she will grow out of this one day. They did some x-rays and blood tests and there was nothing wrong. The Rheumatologist told us she had a score of 5/9 on the Beighton and that meant some of her joints moved more than others. They said that was the only thing different to other kids. They also said it was also ok to give her some days off school, but not too many. We sometimes took days off work as Ellie just looks tired in the morning. I knew if I sent her, we'd get no sleep that night.

Ellie is now 12 years old. I can't remember the last time we all had a full week where we had a full night's sleep. Ellie and her walking up crying is just what we do. My husband and I fight about it sometimes, but our health professionals keep telling us there is nothing seriously wrong with her.

Ellie is also in grade 6 at school and going to high school next year. She still falls asleep in the car on the way home from school some days. Her doctors keep telling us she will grow out of it and suggested we see a podiatrist. She has made some good friends at school, but we are worried about school next year. The campus is HUGE. I'm really worried that it's going to be like Prep all over again. I haven't talked about it with Ellie, but I could see her face at open day.

<u>Questions</u>

How certain are you in the diagnosis of this condition?
How would you explain the pain to Ellia and her family?

> **(Consider What phrases, words and analogies would you usually use in this situation? Describe any non-verbal strategies would you use? How would you discuss daily impact or the future?)**

What management strategies would you recommend for Ellie's pain?

@Monash University, 2023
