# Supplementary material for: Navigating diagnostic uncertainty in children's chronic lower limb pain: A qualitative study of management strategies using vignette‐based focus groups
Source: J Foot Ankle Res. 2025 Mar 5;18(1):e70032. doi: 10.1002/jfa2.70032 (PMC11881608; doi:10.1002/jfa2.70032)
Supplement: Supplementary file 2 — Supplementary Material 2 [file JFA2-18-e70032-s001.pdf]

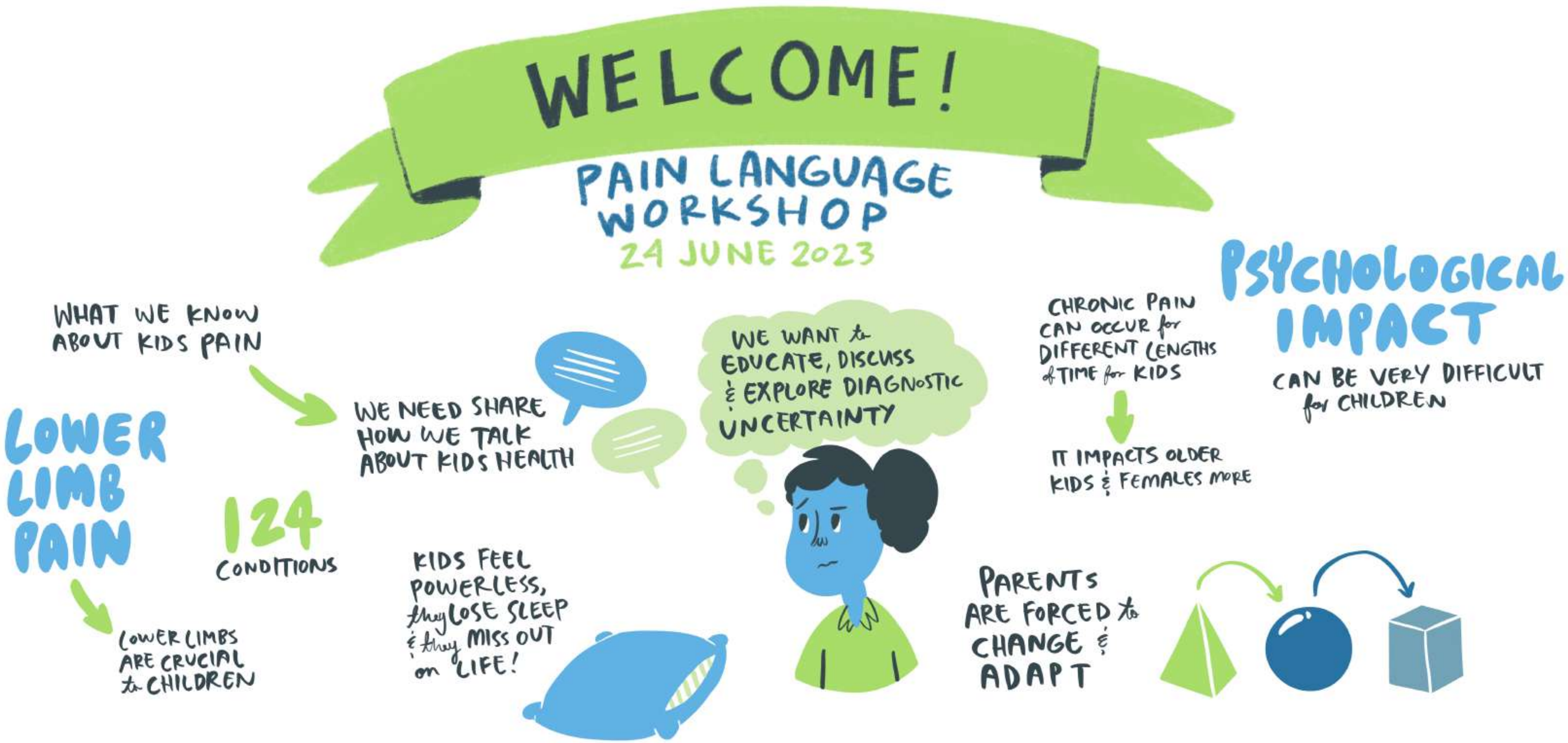

Kid's LEG PAIN

MONASH University

Copyright Jimmy Patch Creative Pty Ltd 2023

PATCH CREATIVE

3 STORIES

FROM KIDS WITH LEG PAIN

PODIATRISTS WERE ASKED ABOUT:

- CERTAINTY of DIAGNOSIS
- HOW THEY WOULD EXPLAIN they PAIN
- WHAT MANAGEMENT STRATEGIES WOULD THEY USE TO HELP WITH the PAIN

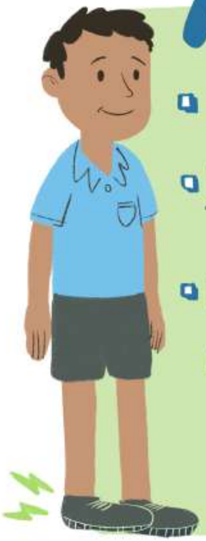

ARCHER

- 11 YEARS OLD
- HAS HEAL PAIN RELATING TO CALCANEAL APOPHYSITIS
- HE IS WORRIED ABOUT the PAIN BECAUSE IT'S MAKING HIM WANT to GIVE UP SOCCER

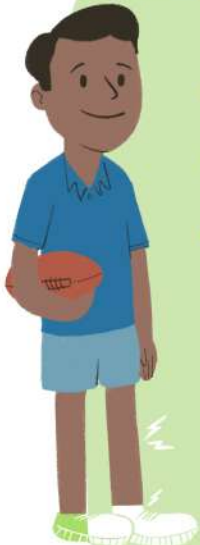

TAMAR

- 11 YEARS OLD
- LOVES AFL
- HAS JIA & EVEN THOUGH IT APPEARS WELL CONTROLLED IT IS IMPACTING HIM IN SCHOOL, HIS SPORT & HOW MUCH HE TELLS HIS FAMILY

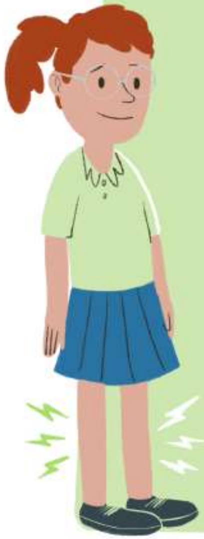

ELLIE

- 12 YEARS OLD
- SHE'S HAD LEG PAIN SINCE SHE WAS 5 THAT MAKES HER CRY AT NIGHT
- SHE HAS SEEN A NUMBER of HEALTH PROFESSIONALS WHO TELL HER SHE WILL GROW OUT of IT
- THIS PAIN IS IMPACTING ALL of THE FAMILY, THEIR SLEEP, THEIR WORK & THEY ARE WORRIED ABOUT the FUTURE

Kid's LEG PAIN

MONASH University

Copyright Jimmy Patch Creative Pty Ltd 2023

PATCH CREATIVE

ARCHER

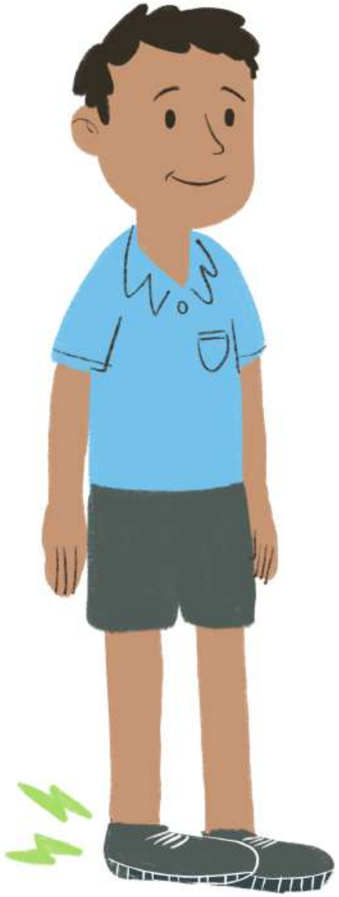

CERTAINTY

- COMMON & MANAGEABLE
- QUITE CERTAIN
- PARTICIPANTS APPEARED CONFIDENT in the DIAGNOSIS AND MANAGEMENT

PAIN EXPLANATION & PAIN MANAGEMENT STRATEGIES

- SELF-RESOLVING
- PAIN FREE IS POSSIBLE
- CHECK THEIR UNDERSTANDING of the PAIN
- REASSURANCE
- NOT USING the WORD 'DISEASE'
- CALCANEAL APOPHYSITIS
- EMPOWER, GROW & BE HAPPY
- DON'T CREATE FEAR
- BEING PERSON CENTRED
- SHARING STORIES ABOUT WHAT WE KNOW
- SHOWING VISUALS of WHAT MIGHT BE CAUSING the PAIN

Kid's LEG PAIN

MONASH University

Copyright Jimmy Patch Creative Pty Ltd 2023

PATCH CREATIVE

TAMAR

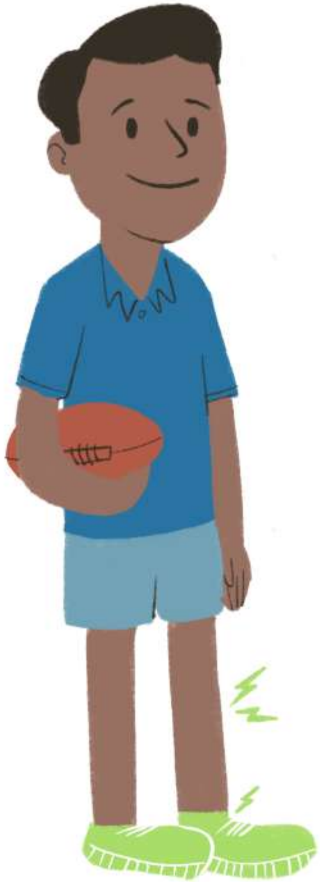

CERTAINTY

- FAIRLY CERTAIN
- DIAGNOSIS CONFIRMED BY MEDICAL
- IT DOES FIT WITH the JIA PROFILE

PAIN EXPLANATION & PAIN MANAGEMENT STRATEGIES

- THE MUM IS the ONE WHO NEEDS SUPPORT. THE CHILD HAS JOY
- YOU CAN'T CHOSE the CONDITION, BUT YOU CAN CHOSE HOW to MANAGE IT
- PHYSIO, EP & HYDRO
- WORK in PARTNERSHIP: ENCOURAGE the KIDS to NOT FEEL RESPONSIBLE for the PARENTS
- DRAWING the PAIN WITH the CHILD
- IT'S OK to HAVE PAIN
- OFFLOADING
- EVERY DAY IS DIFFERENT
- STICKY or JOINT STIFFNESS
- ACKNOWLEDGING that IT IS SCARY
- WHAT MAKES YOU HAPPY?
- MANAGEMENT IS NEVER the SAME for TWO CHILDREN
- WORKING in TEAMS
- SEEING A PAIN SPECIALIST
- HOLISTIC APPROACH
- PERMISSION to FEEL WHAT HE FEELS
- MDT

Kid's LEG PAIN

MONASH University

Copyright Jimmy Patch Creative Pty Ltd 2023

PATCH CREATIVE

ELLIE

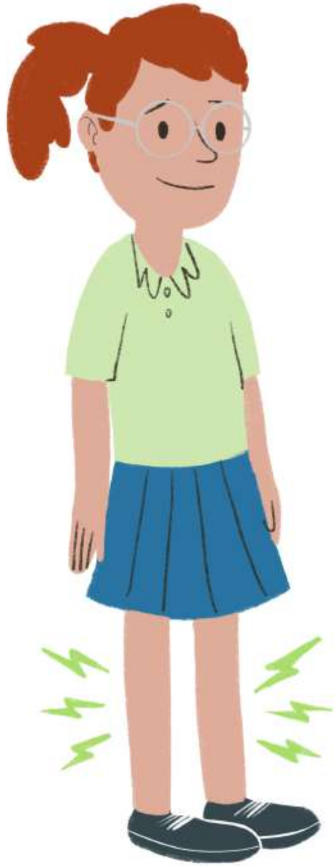

CERTAINTY

- ONGOING SEARCH for A DIAGNOSIS
- DO MORE PEOPLE NEED to BE INVOLVED?
- SHOULD WE ORDER MORE TESTS?
- UNCERTAINTY IS OK

PAIN EXPLANATION & PAIN MANAGEMENT STRATEGIES

- GOOD to RULE OUT the SCARY STUFF
- TEAM for PARENT SUPPORT
- ANALOGIES to HELP EXPLAIN
- MANAGEMENT VIA TEAM to IMPROVE FUNCTION
- THERE'S SO MUCH DOOM HERE
- IT'S OK AS A HEALTHCARE PROFESSIONAL NOT TO KNOW EVERYTHING
- HOW MUCH IS MUM COMMUNICATING?
- EMPOWERING ELLIE to UNDERSTAND HER PAIN
- JUST BECAUSE NOTHING SHOWED UP AT AGE 5 DOESN'T MEAN THERE'S NOTHING NOW
- HOLISTIC LIFESTYLE CHANGES
- MUM'S STRESS; IS IT IMPACTING on LAURA?
- DIETICIAN, ENDOCRINOLOGIST or PAEDIATRICIAN?
- CONNECT PAIN to PAST EXPERIENCES
- IS IT TIME for PAIN or ANXIETY MEDICATION?
- IMPORTANCE of IMPACT on SLEEP

Kid's LEG PAIN

MONASH University

Copyright Jimmy Patch Creative Pty Ltd 2023

PATCH CREATIVE
